# Feasibility and Early Outcomes of Intraoperative Extubation in Pediatric Atrial and Ventricular Septal Defect Repair via Axillary Thoracotomy

**DOI:** 10.21470/1678-9741-2025-0392

**Published:** 2026-09-08

**Authors:** Serkan Seçici, Serkan Terkanlıoglu

**Affiliations:** 1 Department of Cardiovascular Surgery, Manisa Celal Bayar University, Manisa, Turkey; 2 Department of Anesthesiology, Medicana Health Group, Bursa, Turkey

**Keywords:** Enhanced Recovery After Surgery (ERAS), Congenital Cardiac Surgery, Extubation, Minimally Invasive Surgery.

## Abstract

**Introduction:**

This retrospective study evaluated the feasibility, safety, and early recovery outcomes of intraoperative extubation combined with minimally invasive axillary thoracotomy for atrial septal defect (ASD) and ventricular septal defect (VSD) repair in pediatric patients.

**Methods:**

Sixty-one consecutive pediatric patients who underwent ASD (n = 43) or VSD (n = 18) repair via axillary thoracotomy between March 2017 and December 2023 were included. Patients were categorized by extubation timing into intraoperative (operating room [OR] group) and postoperative (intensive care unit [ICU] group). Baseline characteristics, operative variables, and postoperative outcomes were compared.

**Results:**

In the ASD cohort, patients extubated intraoperatively had a shorter hospital stay compared with those extubated in the ICU (2.78 ± 1.24 vs. 3.60 ± 0.99 days, P < 0.001). In the VSD cohort, the OR group included younger and lower-weight patients yet demonstrated a significantly shorter hospital stay (3.56 ± 0.73 vs. 5.78 ± 3.35 days, P = 0.013). Across the entire cohort, on-table extubation was associated with reduced hospital stay (P < 0.001), with no increase in reintubation or major complications.

**Conclusions:**

Intraoperative extubation following axillary thoracotomy for ASD and VSD repair in pediatric patients appears safe and feasible and was associated with a significantly shorter hospital stay, even in lower-weight patients. These findings suggest that intraoperative extubation may be considered as part of enhanced recovery strategies in pediatric congenital heart surgery.

## INTRODUCTION

**Table:** 

Abbreviations, Acronyms & Symbols				
ASD	= Atrial septal defect		LOS	= Length of stay
CPB	= Cardiopulmonary bypass		OR	= Operating room
ERAS	= Enhanced recovery after surgery		OTE	= On-table extubation
ESPB	= Erector spinae plane block		PDA	= Patent ductus arteriosus
ICU	= Intensive care unit		UFTE	= Ultra-fast track extubation
IQR	= Interquartile range		VSD	= Ventricular septal defect

Congenital heart defects are the most common congenital anomalies in children, with atrial septal defect (ASD) and ventricular septal defect (VSD) representing the most frequent lesions requiring surgical repair^[1]^. Surgical outcomes for these defects have achieved excellent standards of safety and quality, with near-universal survival, low morbidity, and minimal need for reoperation. Consequently, the focus of contemporary pediatric cardiac surgery has shifted from improving survival toward minimizing surgical trauma and optimizing recovery and cosmetic outcomes^[2]^. In this context, minimally invasive strategies have been developed to reduce surgical trauma while improving cosmetic and psychosocial outcomes in selected pediatric populations^[3,4]^.

Parallel to these surgical innovations, perioperative management has evolved toward enhanced recovery after surgery (ERAS) protocols, emphasizing early mobilization, reduced sedation, and shorter hospital stays^[5,6]^. Fast-track and ultra-fast track extubation (UFTE) strategies, including on-table extubation (OTE), have become integral components of this paradigm. OTE aligns naturally with the objectives of minimally invasive cardiac surgery by minimizing ventilator dependence, reducing airway complications, and accelerating recovery^[6-8]^. Several studies have shown that OTE can be performed safely in pediatric cardiac surgery, without increasing the risk of reintubation or major complications, while potentially reducing intensive care unit (ICU) utilization and overall length of hospitalization^[9,10]^.

Despite increasing reports of successful OTE in pediatric cardiac surgery, most studies have focused on conventional sternotomy approaches or highly selected patient groups. Evidence regarding OTE in the context of minimally invasive thoracotomy remains limited.

Therefore, this study aimed to evaluate the feasibility, safety, and early recovery outcomes of intraoperative extubation following minimally invasive axillary thoracotomy for ASD and VSD repair in pediatric patients. We hypothesized that when implemented within an ERAS-based perioperative framework, OTE could safely shorten intensive care and hospital stays without increasing postoperative complications, even in small infants.

## METHODS

### Ethical Approval and Study Design

This retrospective cohort study was approved by the Ethical Committee of Health Sciences University Bursa Yuksek Ihtisas Training and Research Hospital (approval number: 2011-KAEK-25 2022/11-06) and conducted in accordance with the Declaration of Helsinki. Owing to the retrospective design, the requirement for informed consent was waived.

The study included consecutive pediatric patients who underwent surgical repair for ASD or VSD via axillary thoracotomy between March 2017 and December 2023. All ASD repairs were performed through an axillary thoracotomy during the study period. For VSD patients, the axillary approach was introduced in August 2019. After implementation, patients weighing < 5 kg or those with associated anomalies such as VSD combined with patent ductus arteriosus (PDA) underwent repair via median sternotomy. As surgical experience increased, the axillary thoracotomy technique was gradually extended to infants weighing < 5 kg.

All procedures were performed by a single cardiac surgeon. Patients who underwent additional procedures beyond septal defect repair or had incomplete medical records were excluded from the study. Patients were categorized by extubation timing into two groups: intraoperative extubation in the operating room (OR) (OR group) and postoperative extubation in the ICU (ICU group).

### Surgical Technique

All operations were performed via a right vertical axillary thoracotomy. After endotracheal intubation, an ultrasound-guided erector spinae plane block (ESPB) was administered for perioperative analgesia. A longitudinal skin incision of 4 - 5 cm was made along the mid-axillary line. Thoracic access was obtained through the third intercostal space for ASD or partial anomalous pulmonary venous return, and through the fourth intercostal space for VSD or partial atrioventricular canal with mitral valve cleft. Central cannulation was achieved through the same incision, even in patients over 30 kg. Cardiopulmonary bypass (CPB) was performed under near-normothermic conditions (34 - 35°C) with full flow.

### Extubation Protocol and Perioperative Care

Until 2020, all patients were routinely extubated in the ICU. Beginning in 2020, the institutional protocol was revised to allow intraoperative extubation in appropriate cases, supported by improvements in surgical and anesthetic practice and by advances in postoperative analgesia. The analgesic protocol consisted of an ESPB in conjunction with scheduled postoperative paracetamol administration.

Extubation in the OR was performed only when predefined readiness criteria were met, including stable hemodynamics, adequate spontaneous ventilation, satisfactory oxygenation, normothermia, and preserved airway protective reflexes. Neuromuscular blockade was reversed with neostigmine and atropine in most cases, whereas sugammadex was selectively used for infants younger than one year or in cases of prolonged relaxation.

Cerebral near-infrared spectroscopy monitoring was not routinely used, as all procedures were short and hemodynamic stability was maintained under continuous arterial monitoring.

### Outcomes

The primary outcome of the study was hospital length of stay (LOS), selected as the most direct indicator of early postoperative recovery. Secondary outcomes included ICU LOS, duration of mechanical ventilation, need for reintubation, and postoperative complications. Safety endpoints were defined a priori as any respiratory or hemodynamic instability requiring intervention, including reintubation. Major complications were defined as events requiring reoperation, prolonged mechanical ventilation (> 24 hours), or significant hemodynamic instability requiring high inotropic support (vasoactive-inotropic score > 10).

### Statistical Analysis

All statistical analyses were conducted using IBM SPSS Statistics for Windows, version 21 (IBM Corp., Armonk, N.Y., USA). Continuous variables were expressed as mean ± standard deviation or median with interquartile range (IQR), depending on distribution assessed by the Shapiro-Wilk test. Categorical variables were expressed as counts and percentages. Between-group comparisons were made using Student’s *t*-test or Mann-Whitney U test for continuous variables, and chi-square or Fisher’s exact test for categorical variables. A two-tailed *P*-value < 0.05 was considered statistically significant.

## RESULTS

A total of 61 pediatric patients underwent surgical repair for ASD and VSD via axillary thoracotomy between March 2017 and December 2023, including 43 with ASD and 18 with VSD. Seventeen VSD repairs were performed via median sternotomy before August 2019. After that date, nine additional patients underwent sternotomy because their body weight was below 5 kg or they had associated anomalies such as VSD combined with PDA. Among these, six patients weighed < 4 kg, with the smallest weighing 2.6 kg.

Among ASD patients, 23 (53.5%) were extubated intraoperatively (OR group) and 20 (46.5%) were extubated postoperatively in the ICU (ICU group). In the VSD cohort, nine patients (50.0%) were extubated in the OR and nine (50.0%) in the ICU.

In the ASD cohort, baseline characteristics were comparable between the groups (Table 1). The sex distribution (OR: 9/14 male/female *vs.* ICU: 7/13, *P*=1.000), median age (60.0 months [IQR 35.5 - 105.5] *vs.* 60.0 months [IQR 40.5 - 72.0], *P* = 0.687), and body weight (18.0 [IQR 12.0 - 26.0] *vs.* 15.5 kg [IQR 14.4 - 20.2], *P* = 0.751) did not exhibit significant differences. Three patients in the OR group and one patient in the ICU group underwent partial anomalous pulmonary venous connection with sinus venous defect. The durations of CPB and cross-clamping were also similar (44.5 ± 6.7 *vs.* 44.4 ± 4.6, *P* = 0.9; and 23.1 ± 4.9 *vs.* 21.4 ± 2.9, *P* = 0.34, respectively). Postoperatively, the OR group experienced a significantly shorter hospital stay (2.78 ± 1.24 *vs.* 3.60 ± 0.99 days, *P* < 0.001) (Figure 1). No reintubation or major complications were observed.

**Table 1:** Comparative perioperative and postoperative outcomes between ASD patients extubated in the OR and ICU.

	Overall	ASD-OR	ASD-ICU	*P*-value
	(n = 43)	(n = 23)	(n = 20)	
Age (months)	60 (36 - 84)	60 (35.5 - 105.5)	60 (40.5 - 72)	0.69
Weight (kg)	17.5 (14 - 24)	18 (12 - 26)	15.5 (14.4 - 20.3)	0.75
Sex (female/male)	27/16	14/9	13/7	1
CPB time (minutes)	44.47 ± 5.75	44.52 ± 6.68	44.4 ± 4.62	0.90
Cross-clamping time (minutes)	22.35 ± 4.13	23.13 ± 4.91	21.45 ± 2.87	0.34
Ventilation time (hours)	-	-	2.0 (1.0-4.0)	-
ICU stay (days)	1.02 ± 0.15	1.0 ± 0	1.05 ± 0.22	0.36
Hospital stay (days)	3.16 ± 1.19	2.78 ± 1.24	3.60 ± 0.99	< 0.001

Values are presented as n, mean ± standard deviation, or median (interquartile range)

ASD=atrial septal defect; CPB=cardiopulmonary bypass; ICU=intensive care unit; OR=operating room

**Fig. 1:**
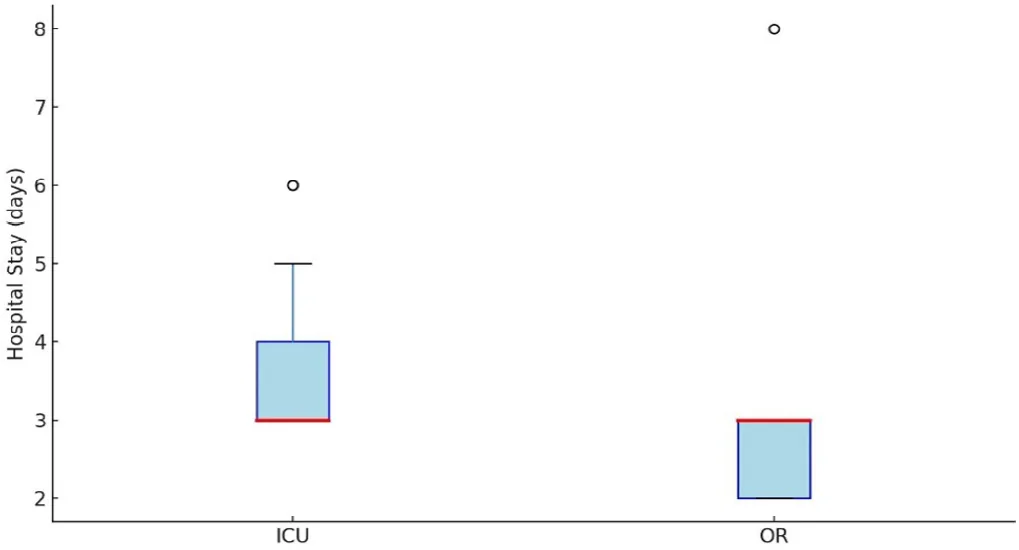
Comparison of hospital stay duration between atrial septal defect patients extubated in the operating room (OR) and in the intensive care unit (ICU).

In the VSD cohort, the sex distribution did not exhibit a statistically significant difference (OR: 5/4 female/male *vs.* ICU: 7/2, *P* = 0.32) (Table 2). Although the mean age was lower in the OR group, this difference was not statistically significant (24.11 ± 16.3 months *vs.* 46.56 ± 29.46 months, *P* = 0.068). Notably, patients who were extubated intraoperatively had significantly lower body weight (7.39 ± 3.04 kg *vs.* 14.08 ± 6.02 kg, *P* = 0.009). The durations of CPB and cross-clamping were comparable between the groups (CPB: 74.22 ± 10.60 minutes *vs.* 71.11 ± 8.10 minutes, *P* = 0.5; cross-clamping: 46.67 ± 7.25 minutes *vs.* 44.67 ± 5.12 minutes, *P* = 0.51, respectively). In the ICU group, only one patient necessitated reintubation due to food aspiration, whereas no reintubation was required in the OR group. The length of ICU stay was similar between the groups (1.11 ± 0.33 *vs.* 1.56 ± 1.01 days, *P* = 0.27). However, the duration of hospital stay was significantly shorter in the OR group than in the ICU group (3.56 ± 0.73 days *vs.* 5.78 ± 3.35 days, *P* = 0.013) (Figure 2).

**Table 2:** Comparative perioperative and postoperative outcomes between VSD patients extubated in the OR and ICU.

	Overall	VSD-OR	VSD-ICU	*P*-value
	(n = 18)	(n = 9)	(n = 9)	
Age (months)	35.33 ± 25.81	24.11 ± 16.25	46.56 ± 29.46	0.068
Weight (kg)	10.73 ± 5.77	7.39 ± 3.04	14.08 ± 6.02	0.009
Sex (female/male)	12/6	5/4	7/2	0.32
CPB time (minutes)	72.67 ± 9.29	74.22 ± 10.60	71.11 ± 8.10	0.50
Cross-clamping time (minutes)	45.67 ± 6.17	46.67 ± 7.25	44.67 ± 5.12	0.51
Ventilation time (hours)	-	-	3.0 (2.0 - 4.0)	-
ICU stay (days)	1.33 ± 0.77	1.11 ± 0.33	1.56 ± 1.01	0.27
Hospital stay (days)	4.67 ± 2.61	3.56 ± 0.73	5.78 ± 3.35	0.013

Values are presented as n, mean ± standard deviation, or median (interquartile range)

CPB=cardiopulmonary bypass; ICU=intensive care unit; OR=operating room; VSD=ventricular septal defect

**Fig. 2:**
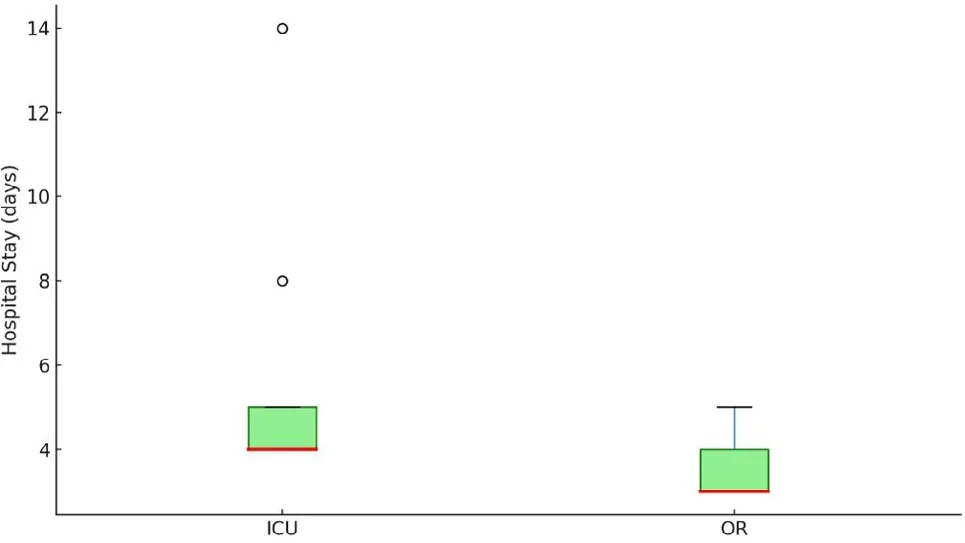
Comparison of hospital stay duration between ventricular septal defect patients extubated in the operating room (OR) and in the intensive care unit (ICU).

Across the entire cohort, ICU stay did not differ significantly between the groups (1.03 ± 0.18 *vs.* 1.21 ± 0.62, *P* = 0.133) (Table 3). However, intraoperative extubation was associated with a significantly shorter hospital stay (3 ± 1.16 *vs.* 4.28 ± 2.22, *P* < 0.001) (Figure 3).

**Table 3:** Comparison of ICU stay and total hospital stay between all patients extubated in the OR and ICU.

	OR	ICU	*P*-value
	(n = 32)	(n = 29)	
Ventilation time (hours)	-	2.0 (1.0-4.0)	-
ICU stay (days)	1.03 ± 0.18	1.21 ± 0.62	0.13
Hospital stay (days)	3 ± 1.16	4.28 ± 2.22	< 0.001

Values are presented as mean ± standard deviation, or median (interquartile range)

ICU=intensive care unit; OR=operating room

**Fig. 3:**
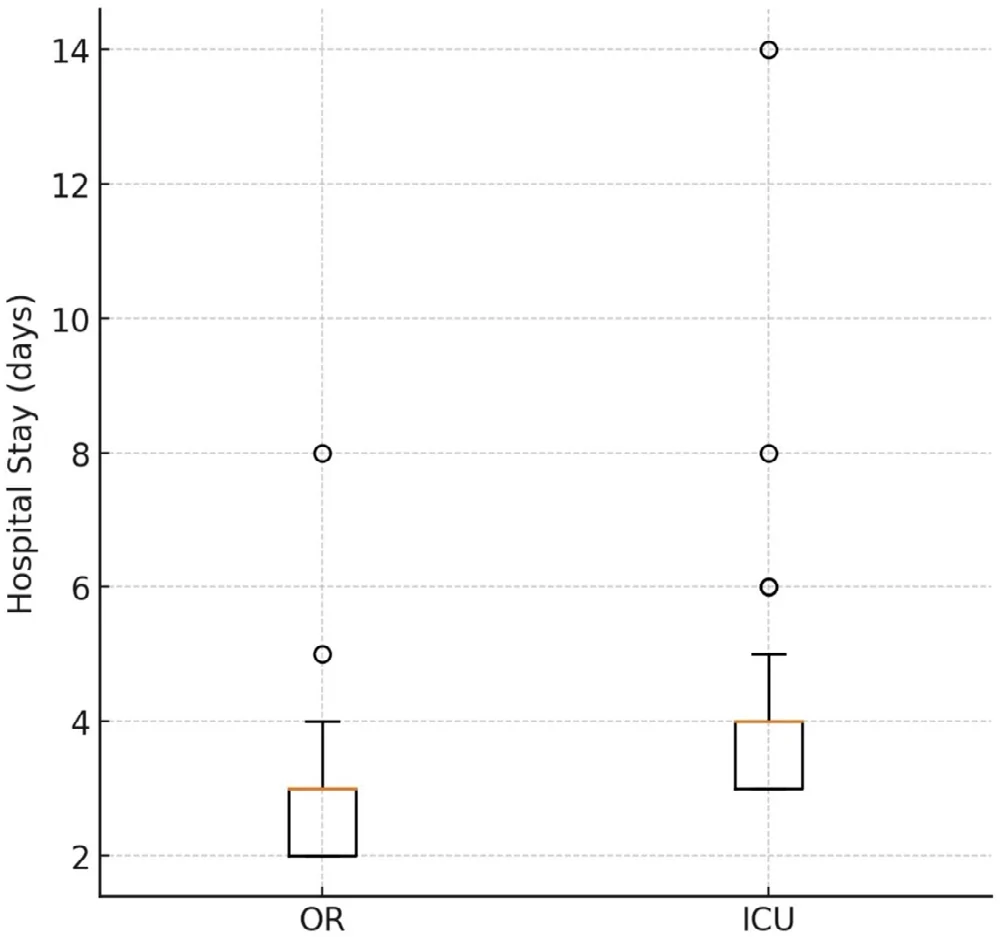
Comparison of hospital stay duration between all patients extubated in the operating room (OR) and in the intensive care unit (ICU).

## DISCUSSION

The present study demonstrates that OTE following minimally invasive axillary thoracotomy for ASD and VSD repair is both safe and feasible in pediatric patients. Importantly, OTE was consistently associated with shorter postoperative hospital stays, even among younger and lower-weight children. These findings are consistent with the principles of ERAS, where fast-track and UFTE strategies are key elements of multidisciplinary perioperative management^[11,12]^. Although early extubation and minimally invasive repair have been previously reported, the present study is among the few to integrate intraoperative extubation with a minimally invasive axillary thoracotomy approach in a homogeneous pediatric cohort operated on by a single surgeon, ensuring uniform perioperative management and minimizing inter-operator variability.

Early extubation after congenital heart surgery aims to reduce ventilator-associated complications, shorten ICU stay, and accelerate postoperative recovery. Historically, concerns regarding airway instability, pulmonary edema, and hemodynamic compromise limited its adoption, especially in smaller infants. However, recent multicenter and single-center studies have demonstrated that when performed under structured protocols and with predefined readiness criteria, early extubation is both safe and beneficial^[9,13]^. Large-scale analyses, including those by Rooney et al.^[14]^ and Faraoni and Ng^[12]^, confirmed that intraoperative or early extubation does not increase reintubation risk while significantly reducing ICU and hospital LOS. In our series, no reintubations occurred after OTE, and patients extubated in the OR had shorter hospitalization times, consistent with prior reports showing faster recovery, reduced nosocomial infections, and less caregiver burden^[15-18]^.

Previous studies have emphasized that the success of early extubation depends not only on timing but also on the perioperative framework in which it is applied. Suominen and Haney highlighted that extubation timing should be viewed as part of a broader ERAS protocol rather than an isolated intervention^[11]^. In our study, OTE was implemented within a minimally invasive surgical context supported by optimized anesthesia and regional analgesia (ESPB), reflecting this comprehensive ERAS approach. The combination of minimal surgical trauma and stable perioperative management facilitated early respiratory independence without compromising safety.

A multimodal analgesia strategy, incorporating regional anesthesia, has repeatedly been identified as a key enabler of safe OR extubation in pediatric cardiac surgery. Among regional techniques, the ESPB has emerged as particularly advantageous because it provides effective opioid-sparing analgesia while preserving respiratory mechanics, thereby facilitating earlier extubation and mobilization^[19,20]^. Evidence suggests that ESPB offers significant analgesic benefits and may yield superior outcomes compared with intravenous opioid regimens, contributing to reduced opioid-related adverse effects^[19,21,22]^. Our findings align with this literature, supporting the concept that effective regional analgesia can underpin successful intraoperative extubation by reducing pain-related respiratory effort and opioid requirements.

Although young age and low body weight have been regarded as relative contraindications for early extubation, emerging evidence challenges this assumption. Murin et al.^[23]^ reported that fast-track extubation could be achieved safely even in infants under 7 kg without an increase in complications. Similarly, in our VSD subgroup, OTE was successfully performed in lower-weight children without adverse events, suggesting that extubation readiness should be based on physiological stability rather than arbitrary thresholds of age or weight.

Beyond physiological outcomes, early extubation improves patient comfort and enhances efficiency of care. Studies showed that reducing mechanical ventilation time leads to fewer pulmonary complications, earlier mobilization, and lower sedation requirements^[12,24]^. Rooney et al.^[14]^ demonstrated that structured early extubation programs yield consistent safety outcomes across diverse centers, underscoring the importance of multidisciplinary coordination. In our experience, standardized readiness criteria and close surgical-anesthetic collaboration were key determinants of success, allowing early transition to spontaneous breathing while maintaining hemodynamic stability.

Economic data further support the clinical rationale for OTE. Holowachuk et al.^[17]^ demonstrated that early extubation reduced total hospitalization costs by approximately USD 13,000 per patient, largely due to shorter ICU stay and decreased ventilation-related resource use. Similarly, Murin et al.^[23]^ showed that fast-track protocols reduced transfusion requirements, inotropic support, and overall hospital LOS. The shorter hospitalization observed in our study cannot be explained by administrative factors, as discharge criteria remained unchanged throughout the study period. Patients were discharged only after meeting objective physiological parameters, including stable hemodynamics, adequate oral intake, afebrile status, and reassuring echocardiographic findings. Therefore, earlier discharge reflects genuine physiological recovery rather than institutional policy.

Incorporating OTE into minimally invasive congenital heart surgery also provides psychosocial benefits. Axillary thoracotomy avoids the visible midline scar associated with sternotomy, improving body image and long-term self-esteem, particularly in children and adolescents^[25]^. While cosmetic outcomes were not directly assessed in our cohort, prior studies indicate that minimally invasive access contributes positively to quality of life, aligning with ERAS goals of minimizing stress and expediting return to normal activity.

The successful implementation of OTE requires institutional commitment and structured team-based practice. Experiences from high-volume centers have demonstrated that standardized readiness criteria and communication between teams are critical to ensuring safety and reproducibility^[26]^. Our findings support the feasibility of embedding OTE within institutional ERAS protocols as a reproducible and beneficial practice for appropriately selected pediatric patients.

### Limitations

This study has limitations. Its retrospective, single-center design limits causal inference and generalizability, and the relatively small VSD subgroup may reduce statistical power. Furthermore, long-term outcomes such as pulmonary function, musculoskeletal development, and cosmetic satisfaction were not evaluated. Nevertheless, the uniform surgical and anesthetic strategy and unchanged discharge criteria enhance the reliability of the results. Prospective multicenter studies with larger cohorts are warranted to validate these findings, define standardized selection criteria, and assess long-term outcomes associated with intraoperative extubation.

## CONCLUSION

Intraoperative extubation following minimally invasive axillary thoracotomy for ASD and VSD repair appears to be a safe and feasible strategy that enhances recovery without compromising patient safety. By reducing hospital stay, optimizing resource utilization, and improving patientand family-centered outcomes, OTE may serve as a valuable component of ERAS-based pediatric cardiac surgery. Further multicenter prospective studies are necessary to confirm these results and facilitate broader adoption within modern congenital cardiac practice.

## Data Availability

The authors declare that the data will be available upon request to the authors.

## Artificial Intelligence Usage

The authors declare use of ChatGPT (OpenAI) for language editing. The content produced by the artificial intelligence tool was revised and edited by the authors as necessary, and they take full responsibility for the content to be published. All scientific content and data analysis were developed by the authors.
